# Structure and physical property correlation in magnesium doped LaFeO_3_ nano perovskites synthesized by the green method

**DOI:** 10.1038/s41598-025-33081-9

**Published:** 2026-01-11

**Authors:** Abdalrahman M. Rayan, Mehawed M. Ahmed, H. A. A. Saadallah, A. A. Azab, Kh. Roumaih, A. M. Abdel Hakeem

**Affiliations:** 1 Physics Department, Faculty of Science, Sohag University, Sohag, 82524 Egypt; 2 Chemistry Department, Faculty of Science, Sohag University, Sohag, 82524 Egypt; 3 Solid State Physics Department, Physics Research Institute, National Research Center, Giza, 12622 Dokki Egypt; 4 Reactor Physics Department, Nuclear Research Center, Egyptian Atomic Energy Authority, Cairo, 13759 Egypt

**Keywords:** Spintronics, Sensing application, Green synthesis, Dielectric properties, Magnetic properties, Materials science, Nanoscience and technology, Physics

## Abstract

In this work, La_1 − x_MgₓFeO₃ (0.0 ≤ x ≤ 0.20) perovskite nanoparticles were successfully synthesized using a green synthesis route assisted by Moringa oleifera leaf extract. The influence of Mg²⁺ substitution on the structural, dielectric, and magnetic properties of LaFeO₃ was systematically investigated. X-ray diffraction and Rietveld refinement revealed a clear phase transition from cubic (Pm-3 m) to orthorhombic (Pnma) symmetry at x ≥ 0.17. Low Mg doping (x = 0.05, 0.10) induced a slight lattice expansion due to oxygen vacancy formation and Fe³⁺ → Fe²⁺ reduction, while higher doping levels led to lattice contraction from ionic size mismatch and structural distortion. Dielectric measurements demonstrated a significant enhancement in dielectric constant and suppression of dielectric loss up to x = 0.15, attributed to improved interfacial polarization and defect-induced conduction. Magnetic analysis via Vibrating Sample Magnetometer (VSM) and electron spin resonance (ESR) confirmed a transition from weak ferromagnetic to strong ferromagnetic behavior with increased Mg content, peaking at x = 0.17 due to enhanced Fe³⁺/Fe⁴⁺ exchange interactions and surface spin effects. These findings highlight the potential of Mg-doped LaFeO₃ nanoparticles for multifunctional applications in energy storage, sensing, and spintronic devices, while emphasizing the environmental and economic advantages of green synthesis approaches.

## Introduction

Perovskite-type oxides with the general formula RTO₃, where R represents a rare-earth element (e.g., La, Ba, etc.) and T is a 3 d transition metal (e.g., Fe, Ni, Co, Mn)^1–4^, have attracted considerable interest due to their remarkable structural flexibility and tunable physical properties. These properties include magnetism, dielectric behavior, ferroelectricity, and catalytic activity. The term “perovskite” originates from the mineral CaTiO_3_, discovered in the Ural Mountains in 1839 and named after the Russian mineralogist Lev Perovski. Since then, synthetic perovskites have emerged as a prominent class of multifunctional materials for various technological applications, including high-temperature superconductivity, piezoelectricity, and gas sensing^5–7^. Among perovskite oxides, lanthanum ferrite (LaFeO₃ or LFO) is particularly significant due to its abundance, low cost, environmental friendliness, and diverse physical properties. LFO exhibits G-type antiferromagnetic ordering with a high Néel temperature (~ 740 K), which arises from Fe^2^⁺-O-Fe³⁺ superexchange interactions. These properties make it a candidate for various applications such as gas sensors^8–12^, hydrogen storage^13^, photo-catalysis^1,14–16^, water purification^17^, and lithium-ion batteries^18^. The physical properties of LaFeO₃ are highly dependent on its particle size, morphology, and crystallinity, which are influenced by the synthesis route. Techniques such as sol-gel processing, solution combustion, and reactive flash sintering offer different advantages in terms of microstructure control, energy efficiency, and scalability^19–21^. Doping is another effective approach to tailor the properties of LaFeO₃. Rare-earth doping (e.g., Gd³⁺, Sm³⁺, Nd³⁺) can distort the perovskite lattice by modifying the Goldschmidt tolerance factor, which enhances the canting of FeO₆ octahedra and induces weak ferromagnetism^22,23^, Transition-metal doping at the B-site (e.g., Co²⁺, Al³⁺) alters the magnetic anisotropy and electrical properties^24,25^ Specifically, Mg²⁺ doping has been shown to improve the dielectric constant and reduce dielectric loss^26^.

The magnetic behavior of LaFeO_3_ nanoparticles deviates significantly from that of their bulk antiferromagnetic (AFM) counterpart, a phenomenon primarily driven by finite-size effects and enhanced surface spin disorder^27,28^. While bulk LaFeO exhibits a G-type AFM structure with a weak residual moment from Dzyaloshinskii-Moriya (DM) interaction, causing a spin-canted state^29,30^, the reduction in particle size to the nanoscale regime introduces an uncompensated magnetic moment at the particle surface. This surface effect, combined with the decreased coordination number and lattice strain, leads to a transition toward weak ferromagnetism or superparamagnetic below a critical size, typically manifesting in a non-zero remnant magnetization M_r_ and coercivity H_c_ at low temperatures. Furthermore, the material often exhibits a blocking temperature T_B_, below which the magnetic moments “freeze” and display a hysteretic response, while above T_B_, they enter a superparamagnetic state characterized by zero M_r_ and H_c_. The competition between the core’s AFM ordering and the surface’s disordered/canted spins is crucial in determining the overall magnetic properties, making LaFeO nanoparticles candidates for applications like multiferroics and high-density magnetic recording^31,32^.

The electrical properties of LaFeO_3_ nanoparticles are characteristic of a p-type semiconductor, with the charge transport mechanism being highly dependent on temperature, oxygen stoichiometry, and nanoscale effects^33,34^. The bulk material’s conductivity is typically low and governed by the hopping of small polarons, specifically the transfer of holes between Fe^3+^ and Fe^4+^ ions, often described by Mott’s Variable Range Hopping (VRH) or Correlated Barrier Hopping (CBH) models^35,36^, depending on the temperature regime. Reducing the particle size to the nanoscale significantly impacts these properties, primarily by increasing the surface-to-volume ratio and enhancing the influence of grain boundaries and oxygen vacancies, which act as donors and modify the Fe^3+^/Fe^4+^ ratio. The material exhibits a negative temperature coefficient of resistance (NTCR), confirming its semiconducting nature, with measured activation energies for conduction typically in the range of 0.2 to 0.4 eV^37,38^. Furthermore, studies employing impedance spectroscopy often reveal a non-Debye type dielectric relaxation^39,40^, dominated at lower frequencies by the Maxwell-Wagner-Sillars (MWS) polarization effect arising from the heterogeneity of the nanocrystalline structure (i.e., grain boundaries versus grain interior)^41,42^. This combination of moderate electrical conductivity and high dielectric constant makes LaFeO_3_ nanoparticles a promising candidate for applications such as solid oxide fuel cell (SOFC) cathodes, sensors multiferroic devices.

The primary aim of this study is to synthesize Mg-doped LaFeO₃ (La_1 − x_MgₓFeO₃; 0.0 ≤ x ≤ 0.20) perovskite nanoparticles using a green, eco-friendly sol-gel method and to systematically investigate the effects of Mg substitution on their structural, dielectric, and magnetic properties. The study focuses on:

- Understanding the structural phase transition from cubic to orthorhombic symmetry induced by Mg doping.
- Evaluating how Mg incorporation influences crystallite size, lattice strain, and Fe-O bonding characteristics.
- Analyzing the frequency-dependent dielectric behavior and the role of Mg ions in enhancing polarization and reducing dielectric loss.
- Examining the evolution of magnetic ordering (from antiferromagnetic to weak/strong ferromagnetism) as a function of Mg concentration.
- Exploring the nature of electron spin interactions through VSM and ESR measurements, with emphasis on double exchange mechanisms and internal magnetic field contributions.

Ultimately, this study aims to provide insight into how Mg doping can be used to tailor the multifunctional properties of LaFeO_3_ nanoparticles for potential applications in magnetic sensors, dielectric devices, and environmentally friendly functional materials.

## Experimental

### Preparation of Moringa oleifera leaf extract

**Fig. 1:**
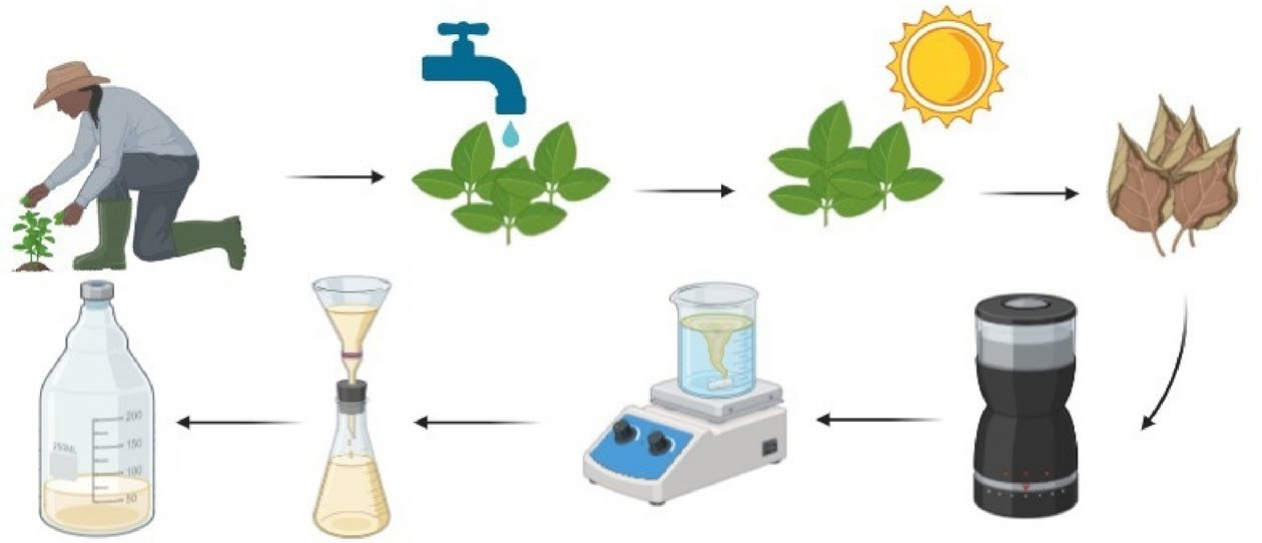
The extraction of fresh Moringa oleifera leaves.

Fresh Moringa oleifera leaves (Family: Moringaceae) were collected from the agricultural farm of Sohag University, Egypt. Mature, healthy leaves were thoroughly washed with tap water, followed by double-distilled water to remove dust and surface contaminants. The cleaned leaves were then sun-dried to eliminate residual moisture and subsequently ground into a fine powder using a mechanical grinder. A 5 g sample of the leaf powder was soaked in 100 mL of double-distilled water and heated at 60–70 °C for 60 min using a magnetic stirrer-hotplate until the solution changed color from clear to light yellow. The resulting extract was filtered several times through Whatman No. 1 filter paper and stored in sterile bottles at 4 °C for future use. Figure 1 illustrates the extraction process of the fresh Moringa oleifera leaves.

### Synthesis of La_1 − x_Mg_x_FeO_3_ nanoparticles

La_1 − x_Mg_x_FeO₃ nanoparticles with doping concentrations of X = 0.0, 0.05, 0.10, 0.15, 0.17, and 0.20% were synthesized using a green sol-gel method. High-purity precursors La(NO_3_)_3_, Fe(NO_3_)_3_.9H₂O, and Mg(NO_3_)_3_.6H₂O (each at 0.0125 mol) were dissolved separately in deionized water and then mixed in a 1000 mL beaker. Natural capping agents derived from flour (dissolved in 20 mL hot deionized water) and 100 mL of Moringa oleifera extract were added dropwise into the mixture while stirring continuously at 70 °C for 3 h.

**Fig. 2:**
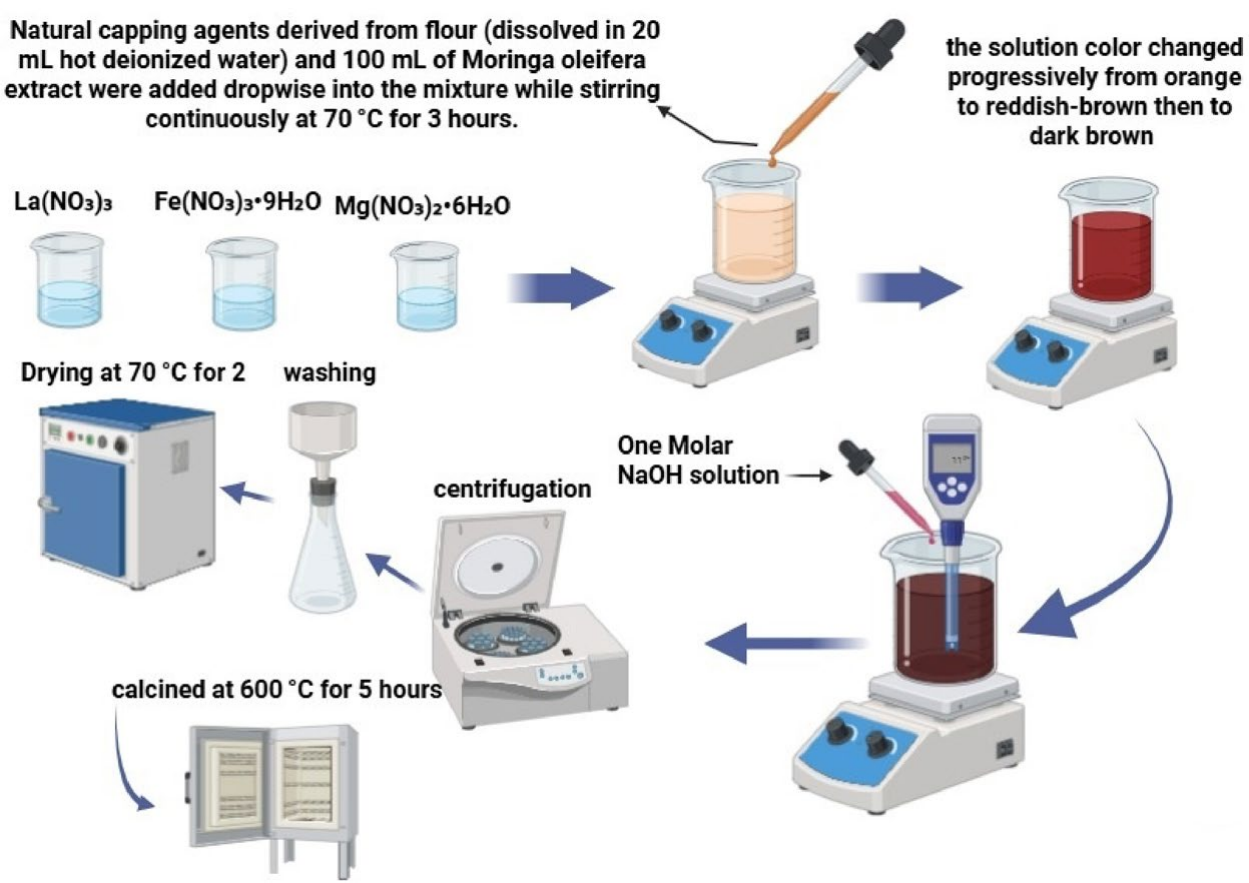
The preparation method of La_1 − x_Mg_x_FeO_3_.

During the reaction, the solution color changed progressively from orange to reddish-brown, then to dark brown. A 1 M NaOH solution was added dropwise to maintain the pH at approximately 11, facilitating the precipitation of the product. The resulting nanoparticles were collected by centrifugation, washed repeatedly with deionized water, and dried at 70 °C for 2 h. The dried paste was placed in a ceramic crucible and calcined at 600 °C for 5 h. A fine black powder was obtained and stored for subsequent characterization. Figure 2 illustrates the preparation method.

## Results and discussion

### Structural analysis

X-Ray Diffractometry (XRD) data of the La_1 − x_Mg_x_FeO₃ nanoparticles were collected with a D8 Advance with DAVINCI design (Bruker, Germany), using as X-ray source the Cu Kα radiation (wavelength λ = 1.5418 Å), at 40 kV and 40 mA, a 2θ range of 20–80°, a step size of 0.02°, and a time/step of 0.6 s. A Si zero-background sample holder was used, operated by DIFFRAC. Measurements Center Version V7.3.0 (32-bit) software, while the assignment of peaks was based on the Powder Diffraction Files (PDF).

**Fig. 3:**
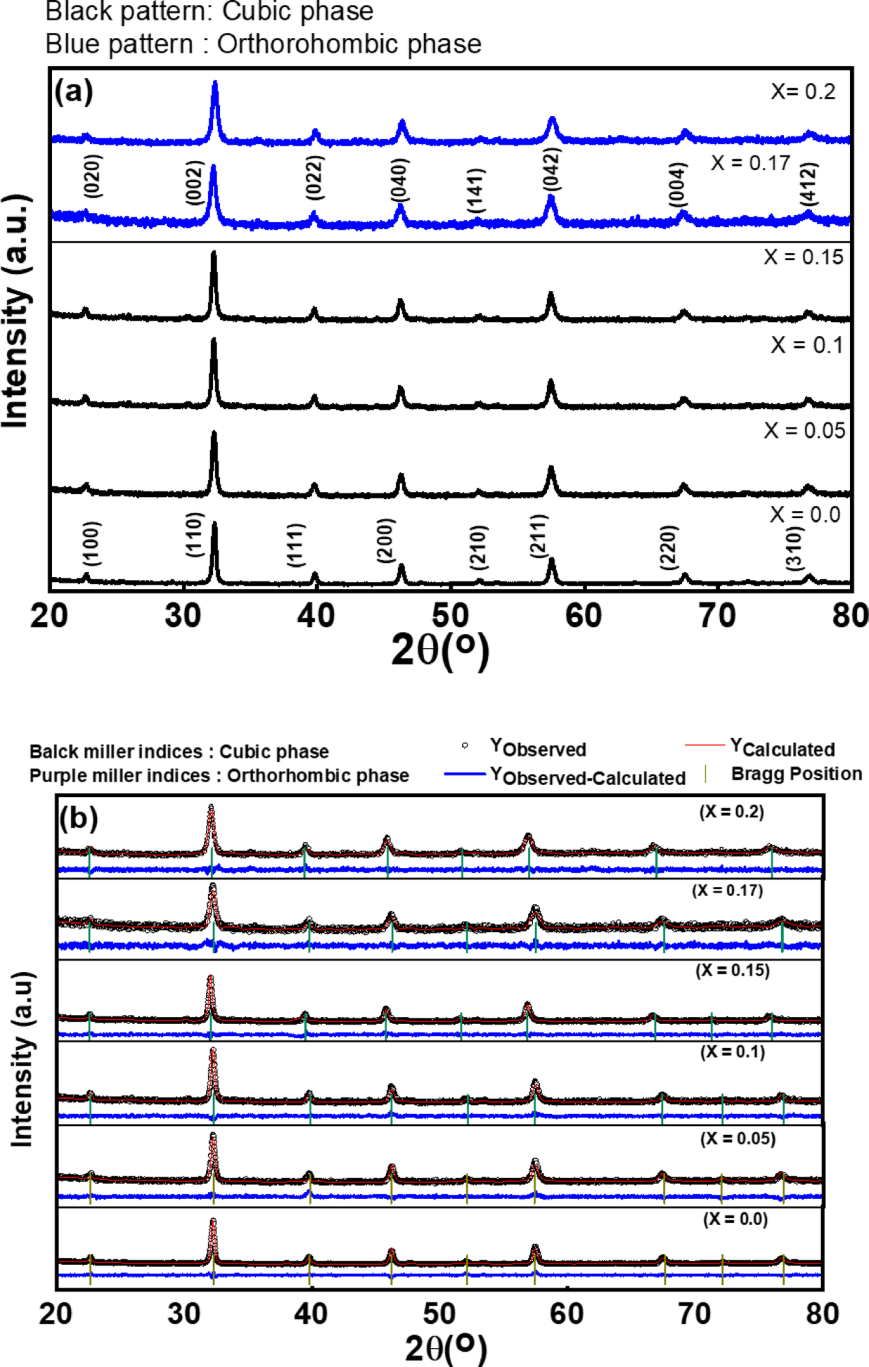
(**a**) Powder XRD pattern, (**b**) rietveld refinement analysis for La_1 − x_Mg_x_FeO_3_ samples.

X-ray diffraction (XRD) analysis was performed to investigate the phase composition of magnesium-doped lanthanum ferrite (La_1 − x_Mg_x_FeO₃). Six different stoichiometries were studied, corresponding to x = 0.0, 0.05, 0.10, 0.15, 0.17, and 0.20 at%. The XRD patterns and corresponding Rietveld refinement profiles for La₁₋ₓMgₓFeO₃ nanoparticles are presented in Figure. 3(a) and Figure. 3(b), respectively. As shown in Fig. 3a, the sharp and well-defined diffraction peaks indicate a high degree of crystallinity in all samples. Phase identification was carried out using X’pert High Score Plus software. Samples with x = 0.0, 0.05, 0.10, and 0.15 matched well with the standard JCPDS card No. 01–075-0541, which corresponds to a cubic perovskite structure (space group Pm-3 m) of LaFeO₃^43^ In this cubic structure, each La³⁺ ion is coordinated by twelve O²⁻ ions, forming LaO₁₂ cuboctahedra. These polyhedra share corners with twelve other LaO₁₂ cuboctahedra, faces with six, and faces with eight neighboring FeO₆ octahedra. When the Mg doping level exceeded 0.15 at% (i.e., x ≥ 0.17), structural distortion occurred, and the symmetry shifted from cubic to orthorhombic. This phase transition corresponds to an orthorhombic perovskite structure of LaFeO₃ (space group Pnma), consistent with JCPDS card No. 96–152-6451^26,44,45^. Importantly, all synthesized samples were confirmed to be single-phase with no detectable secondary phases or impurities. However, a noticeable peak shift toward higher 2θ angles (right shift) was observed as the Mg content increased, indicating lattice contraction. This can be attributed to the substitution of La³⁺ (ionic radius 1.06 Å) with smaller Mg²⁺ ions (ionic radius 0.72 Å), as well as the influence of the specific synthesis route employed^43^.

Rietveld refinement was conducted to confirm the crystal structure and to optimize the structural parameters, as illustrated in Fig. 3b. The refinement results show excellent agreement between the observed and calculated diffraction patterns, indicating high phase purity and reliable structural modeling.

Crystallite size and lattice microstrain were calculated for the synthesized samples, taking into account the instrumental broadening effect of the diffractometer. The total broadening of the XRD peaks was estimated using the following Equation^46–48^:

1 $$\beta _{{(corrected)}} = {\text{ }}\left( {\beta ^{2} _{{refined}} - {\text{ }}\beta ^{2} _{{diffractometer}} } \right)^{{1/2}}$$

Here, β corrected represents the full width at half maximum (FWHM) corrected for instrument-related broadening, β refined is the FWHM obtained from Rietveld refinement, and β diffractometer corresponds to the instrumental contribution to the peak broadening. The corrected FWHM values are listed in Table 1.

These corrected FWHM values were used to calculate the crystallite size and microstrain using the following equation states the Halder-Wagner model^49^.

2 $$\:({\frac{\beta\:}{2tan\theta\:})}^{2}=\:\frac{1}{4}\:\frac{k\lambda\:}{D}\:\frac{\beta\:}{tan\theta\:\:sin\theta\:}+({2\epsilon\:)}^{2}$$

**Fig. 4:**
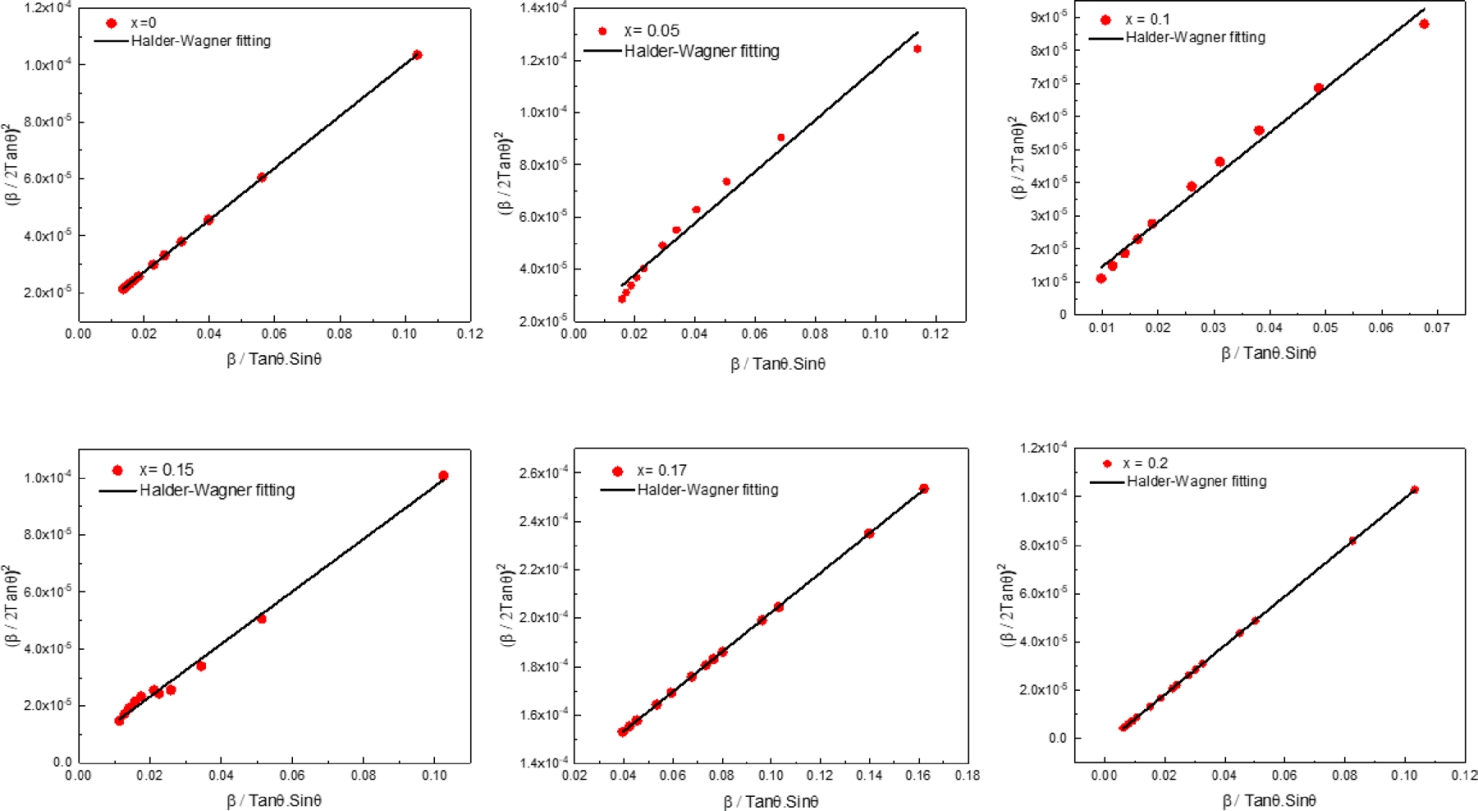
Halder-Wagner fitting for La_1 − x_Mg_x_FeO_3_ samples.

**Fig. 5:**
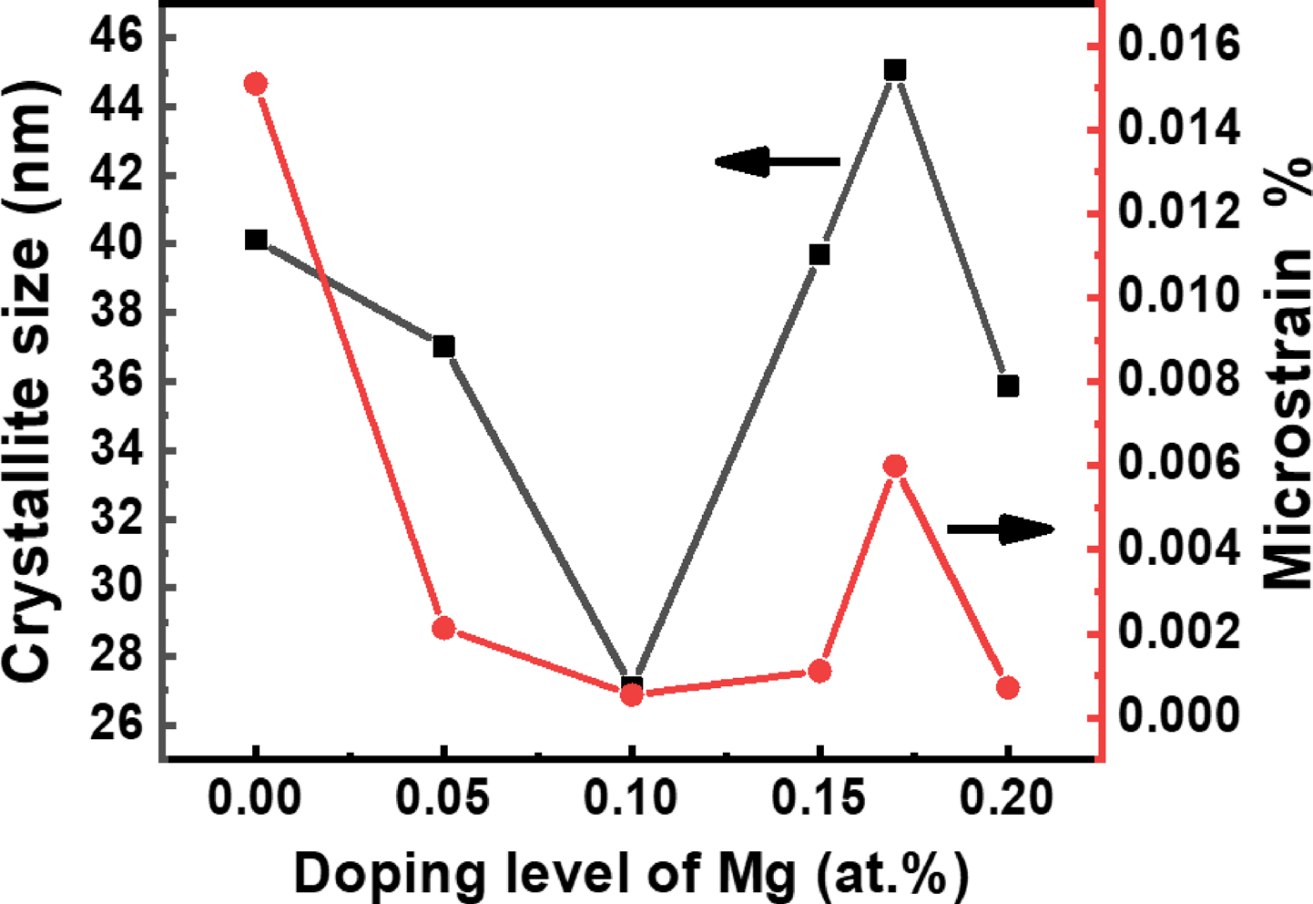
Crystallite size and microstrain for La_1 − x_Mg_x_FeO_3_ samples.

Where $\:\beta\:$ is the full width at half maximum (in radians) after correction, K is a shape factor usually taken as 0.95, $\:\lambda\:$ is the intrinsic wavelength of CuK_α,_ taken as 1.5406 Å,$\:\theta\:$ is the angular position in degrees, D is the crystallite size, and $\:\epsilon\:\:$ is the root mean square of the isotropic strain of Plotting the term $\:\frac{\beta\:}{4tan\theta\:\:sin\theta\:}$ on the x-axis and $\:({\frac{\beta\:}{2tan\theta\:})}^{2}$ on the y-axis, the slope is $\:\frac{k\lambda\:}{D}$ and the intercept is $\:({2\epsilon\:)}^{2}$. It is shown in Fig. 4. The resulting crystallite sizes (C.S) and microstrain values are illustrated in Fig. 5. The crystallite sizes ranged from 27 to 45 nm, while the effective microstrain values were in the range of 0.001–0.016%. These extremely low microstrain values confirm the successful formation of a highly ordered cubic perovskite structure for Mg doping levels of x = 0.0, 0.05, 0.10, and 0.15 at%, and an orthorhombic perovskite structure for x = 0.17 and 0.20 at%. Additionally, any contribution of microstrain to structural distortion can be considered negligible.

**Table 1:** The β _(corrected)_ results for La_1 − x_Mg_x_FeO_3_ samples.

(hkl)	β_corrected_				(hkl)	β_corrected_	
	X = 0	X = 0.05	X = 0.1	X = 0.15		X = 0.17	X = 0.2
(100)	0.004069	0.004456	0.004436	0.004017	(020)	0.006377	0.004069
(110)	0.004490	0.005486	0.005416	0.004110	(002)	0.008278	0.004055
(111)	0.004878	0.006190	0.005984	0.004212	(022)	0.009875	0.004043
(200)	0.005252	0.006752	0.006371	0.004322	(040)	0.011329	0.004032
(210)	0.005622	0.007237	0.006647	0.004833	(141)	0.012711	0.004020
(211)	0.005995	0.007672	0.006839	0.005540	(042)	0.014076	0.004009
(220)	0.006769	0.008456	0.007008	0.006457	(004)	0.016803	0.003986
(310)	0.007608	0.009181	0.006857	0.006967	(412)	0.019888	0.003961

According to the XRD Rietveld refinement results, Table 2 presents the goodness-of-fit (χ²), lattice constants, unit cell volume, space group, crystal structure, and R-factors for the synthesized samples. The data confirm a clear phase transition in the crystal structure as a function of Mg doping. The samples with doping concentrations of x = 0.0, 0.05, 0.10, and 0.15 at% Exhibit a cubic perovskite-like structure, whereas samples with higher doping levels (x = 0.17 and 0.20 at% % %) transition to an orthorhombic perovskite-like structure. The goodness-of-fit parameter (χ²), which reflects the agreement between the observed and calculated profiles (R_wp_ vs. R_exp_), has values close to 1, indicating a high degree of precision in the refinement.

For the cubic samples, at low Mg²⁺ doping levels (x = 0.05 and 0.10 at%), a slight increase in the lattice constants and unit cell volume was observed with increasing Mg content, reaching a maximum value of 60.6780 Å³. This expansion can be attributed to the substitution of Mg²⁺ for La³⁺, which introduces a charge imbalance due to the lower valence state of Mg²⁺. In response, the LaFeO₃ lattice may undergo two primary compensatory mechanisms: the creation of oxygen vacancies, which typically leads to lattice expansion, or the reduction of Fe³⁺ to Fe²⁺, whose larger ionic radius (~ 0.78 Å vs. ~0.645 Å for Fe³⁺) also contributes to lattice expansion^50,51^. However, with a further increase in Mg²⁺ concentration (x = 0.15 at%), a slight decrease in both the lattice constants and unit cell volume was observed, reaching a minimum of 60.4898 Å³. At this stage, the dominant effect becomes the substitution of the smaller Mg²⁺ ions into La³⁺ sites in higher proportions. The structure can no longer fully compensate for the charge imbalance through oxygen vacancies or Fe valence changes alone. This results in a net lattice contraction, driven by the reduced average ionic radius at the A-site. Furthermore, increased structural distortion may promote a symmetry transition from cubic to orthorhombic, which is generally accompanied by a contraction in unit cell volume. This observed reduction is attributed to lattice shrinkage arising from the ionic size mismatch between La³⁺ (1.06 Å) and Mg²⁺ (0.72 Å)^52,53^. In the orthorhombic phase region (x = 0.17–0.20 at%), the lattice constants continued to decrease slightly, and the unit cell volume exhibited a modest reduction from 242.76 to 242.04 Å³. This trend further confirms the occurrence of lattice contraction as a result of Mg substitution. These findings are consistent with previously reported results^52,53^.

**Table 2:** Lattice constant, unit cell volume, space group, crystal structure, and Rietveld refinement factors of La_1 − x_Mg_x_FeO_3_ samples.

Doping level	χ^2^	Lattice constants	Unit cell volume	Space group	Crystal structure	*R*- factors
X = 0.0	1.76	a = b = c = 3.926441(Å)	60.5337(Å^3^)	Pm-3 m	Cubic	R_p_ : 35.7 R_wp_: 21.3 R_exp_: 16.09 Bragg R-factor: 3.92
		α = β = γ = 90.000^o^				
X = 0.05	1.41	a = b = c = 3.929558(Å)	60.6780(Å^3^)	Pm-3 m	Cubic	R_p_ : 56.9 R_wp_: 32.9 R_exp_: 27.63 Bragg R-factor: 7.46
		α = β = γ = 90.000^o^				
X = 0.1	1.34	a = b = c = 3.926543(Å)	60.5384(Å^3^)	Pm-3 m	Cubic	R_p_ : 58.9 R_wp_: 31.3 R_exp_: 26.99 Bragg R-factor: 6.76
		α = β = γ = 90.000^o^				
X = 0.15	1.56	a = b = c = 3.925491(Å)	60.4898(Å^3^)	Pm-3 m	Cubic	R_p_ : 60.5 R_wp_: 32.8 R_exp_: 26.19 Bragg R-factor: 3.62
		α = β = γ = 90.000^o^				
X = 0.17	1.26	a = 5.572032(Å)	242.0487(Å^3^)	Pnma	Orthorhombic	R_p_ : 103 R_wp_: 48.6 R_exp_: 43.01 Bragg R-factor: 91.3
		b = 7.840774(Å)				
		c = 5.540260(Å)				
		α = β = γ = 90.000^o^				
X = 0.2	1.26	a = 5.583467(Å)	241.7666(Å^3^)	Pnma	Orthorhombic	R_p_ : 70.6 R_wp_: 37.7 R_exp_: 33.56 Bragg R-factor: 46.6
		b = 7.833086(Å)				
		c = 5.527890(Å)				
		α = β = γ = 90.000^o^				

**Fig. 6:**
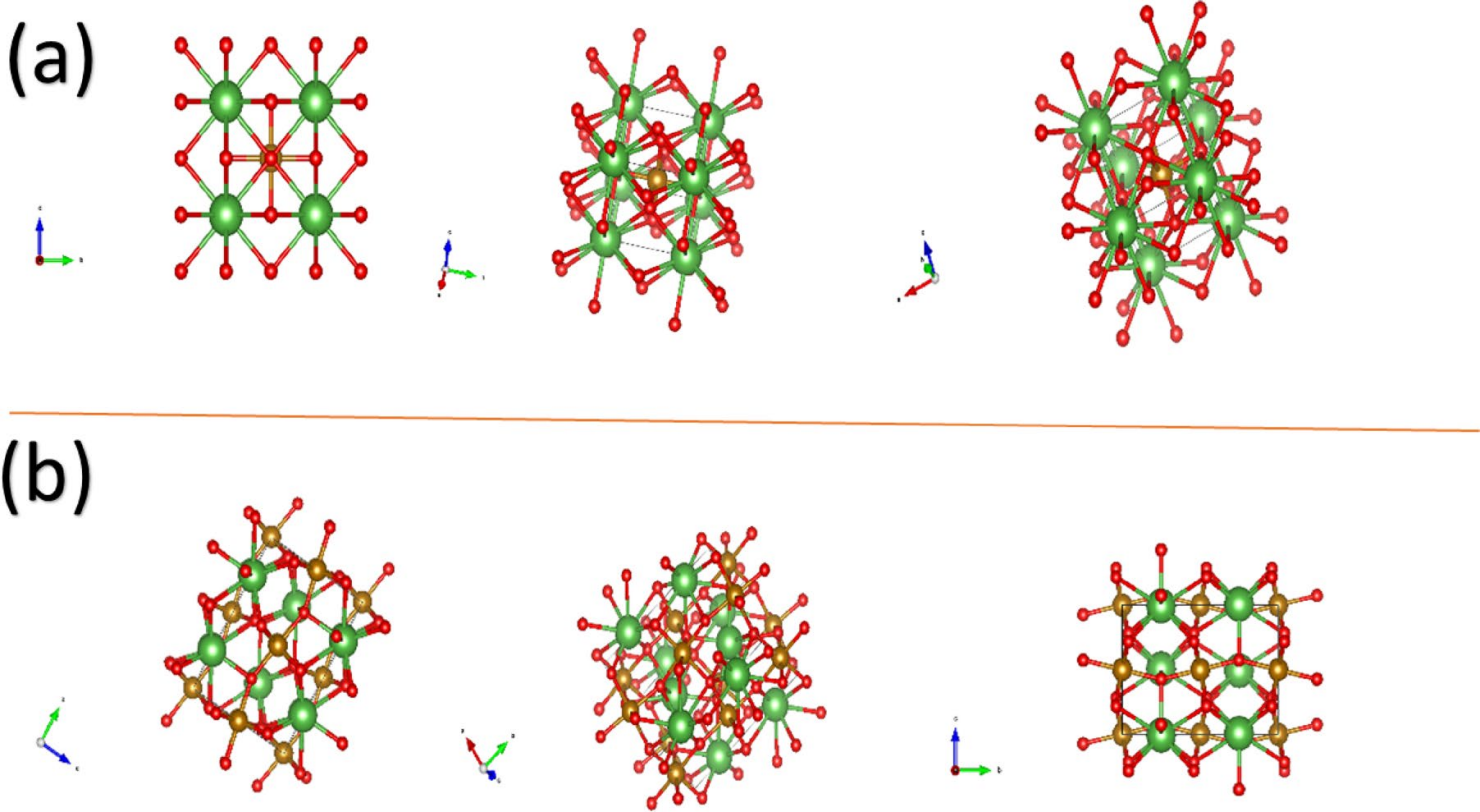
(**a**) The simulated crystal structure of the first four ratios (x = 0.0, 0.05, 0.1, 0.15), owing cubic crystal structure, and (**b**) for x = 0.17, 0.20 at% owing to an orthorhombic crystal structure.

The simulated crystal structures of the cubic and orthorhombic perovskite-like phases of LaFeO₃ were generated using VESTA software. Figure 6a,b illustrates the unit cell configurations for various Mg doping concentrations. The cubic structures are shown in Fig. 6a for x = 0.0, 0.05, 0.10, and 0.15 at%, while the orthorhombic structures corresponding to x = 0.17 and 0.20 at% are presented in Fig. 6b. These simulations demonstrate a phase transformation from the ideal cubic perovskite structure to an orthorhombic structure as the Mg doping level increases beyond x ≥ 0.17 at%. This structural transition is attributed to Jahn-Teller distortion, which becomes more significant at higher dopant concentrations^54–56^.

The bond lengths and bond angles between Fe/Mg and O in the cubic and orthorhombic structures of LaFeO_3_ nanoparticles are presented in Tables 3 and 4, respectively. The observed values indicate an increase in both bond lengths and bond angles, suggesting that Mg substitution significantly influences the bonding and dynamic distortion of the FeO_6_ octahedral^26,57^.

**Table 3:**
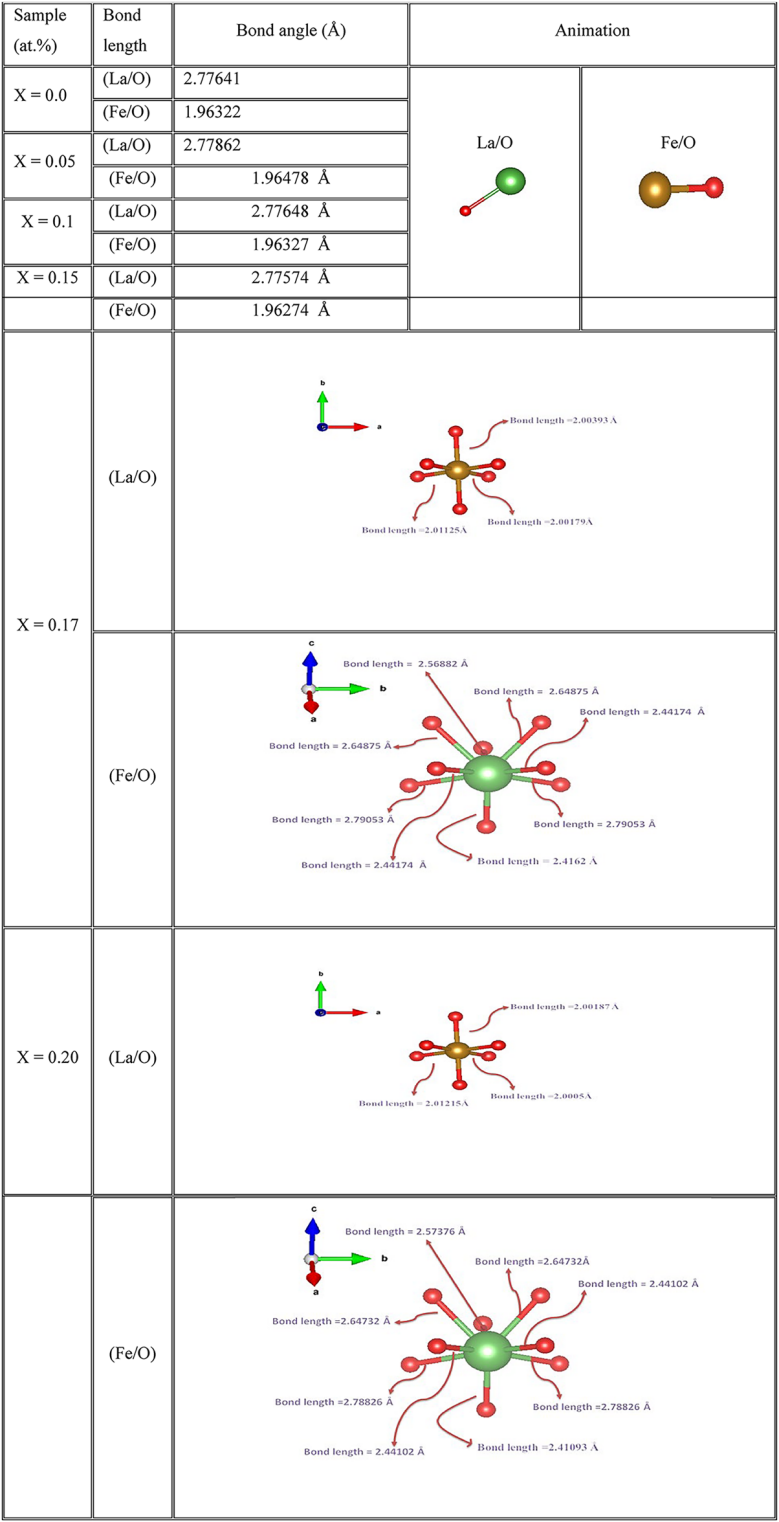
The bond lengths of the cubic and orthorombic ratios for La_1-x_Mg_x_FeO_3_ samples.

**Table 4:**
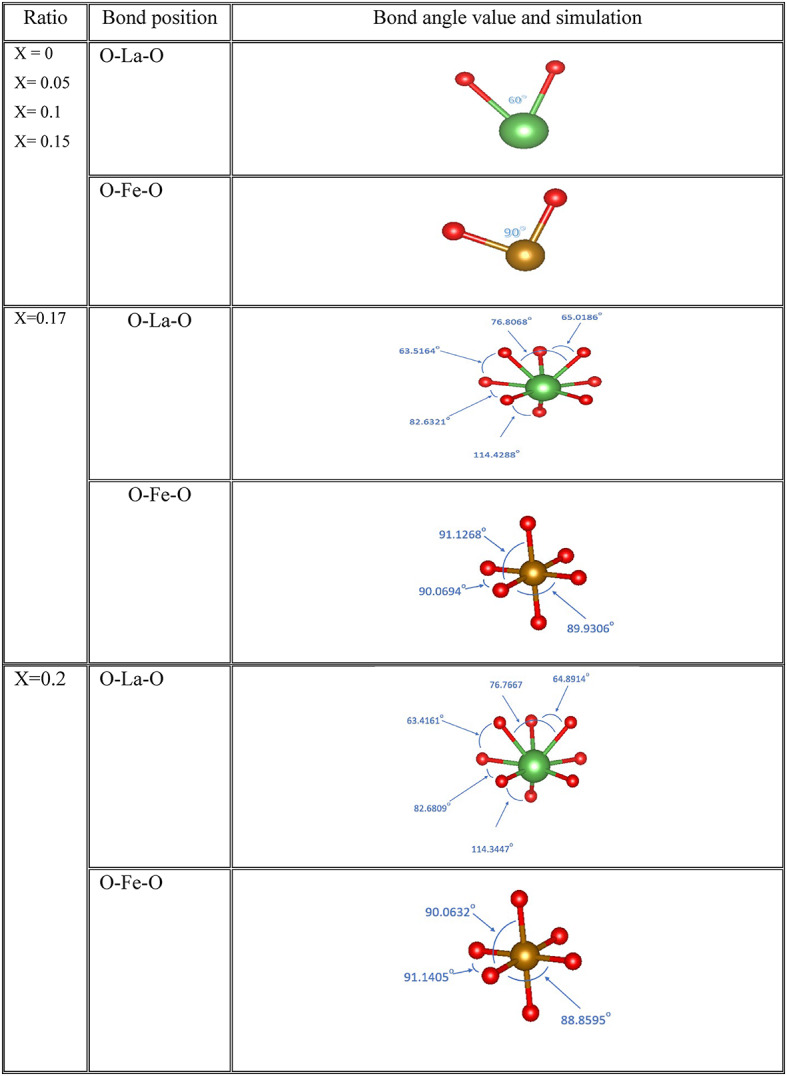
The bond angles in cubic and orthorhombic ratios of La1-xMgxFeO3 samples.

## Dielectric properties

**Fig. 7:**
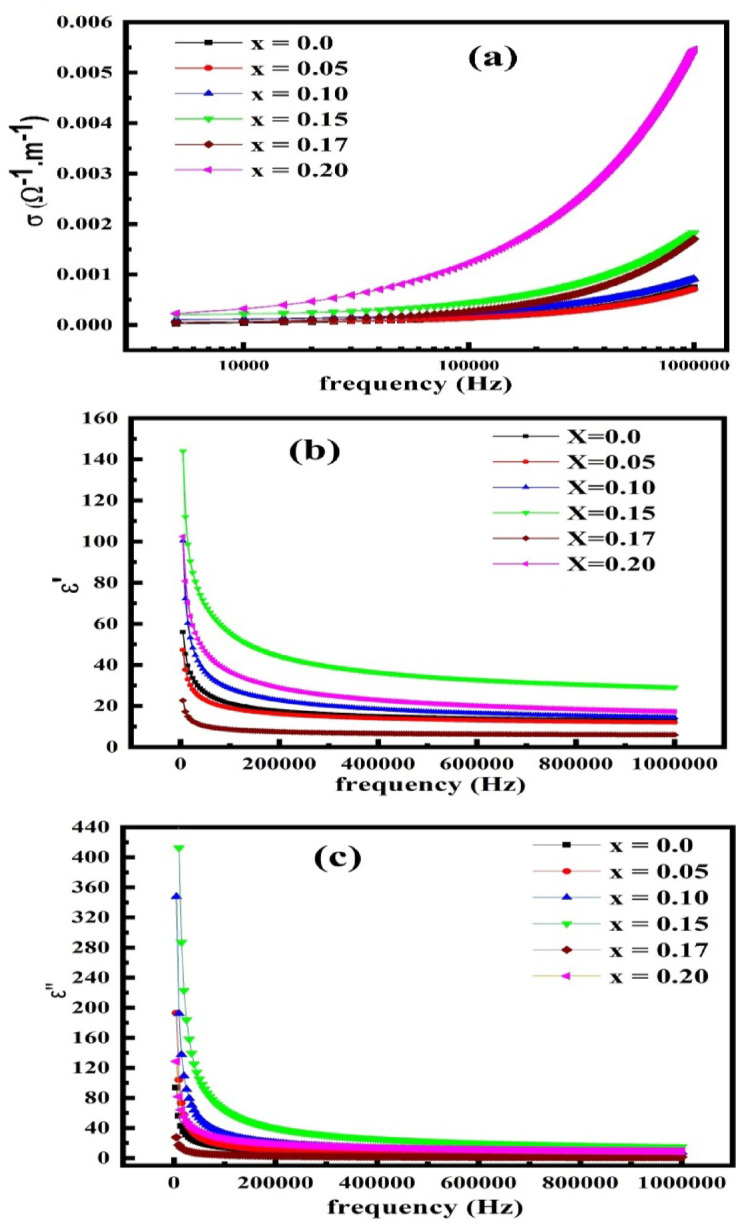
(**a**) AC conductivity, (**b**) Dielectric constant, (**c**) Dielectric loss factor for La_1 − x_Mg_x_FeO_3_ samples.

Figure 7 illustrates the frequency dependence of (a) AC conductivity, (b) dielectric constant, and dielectric loss factor for La_1 − x_Mg_x_FeO₃ samples with x = 0.0, 0.05, 0.10, 0.15, and 0.20 at% at room temperature. The AC conductivity shows a plateau-like region at low frequencies, corresponding to the DC conductivity (σ_dc_) up to a characteristic frequency (νc)^58–60^. Beyond this region, the AC conductivity follows Jonscher’s universal power law at higher frequencies. The overall frequency-dependent conductivity (σ) can be described by the relation^61,62^:

3 $$\sigma {\text{ }} = {\text{ }}\sigma _{{dc}} + {\text{ }}\sigma _{{ac}}$$

where σ_dc_ is the conductivity at zero frequency, and σ_ac_ is the purely frequency-dependent component. This model is commonly used to describe relaxation phenomena in dielectric materials.

The AC conductivity component (σ_ac_) as a function of angular frequency (ω = 2πf) follows Jonscher’s power law^63^:

4 $$\sigma _{{ac}} = {\text{ }}A{\text{ }}\omega ^{s}$$

The primary mechanism of electrical conductivity in metal oxides is electron hopping between ions with different oxidation states^64–66^. These oxides typically contain mixed-valent cations, allowing for electron transfer from, for instance, Fe²⁺ to a neighboring Fe³⁺ ion, thereby altering their oxidation states. This charge transfer results in a net displacement of electrons, contributing to electrical conductivity^65^.

Koop’s theory further explains the frequency-dependent behavior of conductivity in such oxides. According to this model, the material consists of highly conductive grains separated by poorly conducting grain boundaries. At higher frequencies (f > 10⁴ Hz at room temperature), the electrical response becomes more dominated by the grains, leading to increased conductivity^65,67^.This dispersion in conductivity is attributed to the enhanced hopping frequency of charge carriers across the grain boundaries. Moreover, the observed increase in both AC and DC conductivity at high frequencies may be partially due to enhanced tunneling probability of charge carriers, which is facilitated by thermal oscillations of localized sites^68^. As shown in Fig. 7a, conductivity increases with rising Mg content, indicating that magnesium doping enhances carrier mobility. Bougoffa et al.^69^synthesized La₀.₈Ca₀.₁Pb₀.₁Fe_1 − x_MgₓO₃ (x = 0.0, 0.1, 0.2 at%) and reported that incorporating magnesium ions at concentrations below 10% led to a decrease in resistivity and a corresponding increase in conductivity. Conversely, U. Hanifah et al.^26^ prepared La_1 − x_Mg_x_FeO₃ (x = 0.0, 0.1, 0.2, 0.3 at%) using a sol-gel method followed by sintering, and observed a decrease in conductivity with increasing Mg content. This discrepancy may be due to differences in synthesis conditions, crystal structure evolution, and defect formation behavior.

Figure 7b illustrates the variation of the dielectric constant with frequency (10 Hz to 1 MHz) at room temperature. The Figure illustrates how the dielectric constant falls as frequency rises and reaches a constant value at higher frequencies. The variation indicates the dispersion caused by Maxwell-Wagner type interfacial polarization, consistent with Koop’s phenomenological theory. The behavior of current samples is the normal behavior of oxides and ferrites^70–74^. The mechanisms of electric conduction and dielectric polarization in ferrites are similar^65^. Polarization is determined by the local displacement of electrons in the direction of an applied electric field, which results from the electron exchange between ions with different oxidation states^73–75^. The dielectric behavior of the perovskites and the conduction mechanism are strongly correlated by Iwauchi and Ikeda^76^. The polarization significantly diminishes with an increase in frequency and attains a constant value because, beyond a certain frequency of the external field, the electron exchange between ions with different oxidation states cannot keep pace with the alternating field^77,78^. The high dielectric constant values noted at lower frequencies can be elucidated through Koops’ phenomenological theory. Their presence is attributed to the dominance of ions with different valences, interfacial dislocation pile-ups, grain boundary defects, and oxygen vacancies, etc^73,74^. The reduction in dielectric constant with frequency occurs because any species contributing to polarizability tends to lag behind the applied field at elevated frequencies. The decrease in dielectric constant with frequency is because any species contributing to polarizability is bound to lag behind the applied field at higher frequencies. The addition of Mg ion enhanced the dielectric constant up to x = 0.15 and then fell after that. Figure 7c depicts the relationship between the imaginary component of the dielectric constant (dielectric loss) and the frequency of La_1 − x_Mg_x_FeO_3_ at ambient temperature. Dielectric loss diminishes as frequency increases. The values of the imaginary component rise at low frequencies as a result of material conductivity and the movement of free charge carriers^79^. Figure c illustrates that the dielectric loss factor has the same behavior as the dielectric constant, where the dielectric loss factor increases with Mg content to reach the maximum loss at x = 0.15 and then decreases at x = 0.17 and 0.20 at%.

## Magnetic properties

### Magnetic hysteresis loops

**Fig. 8:**
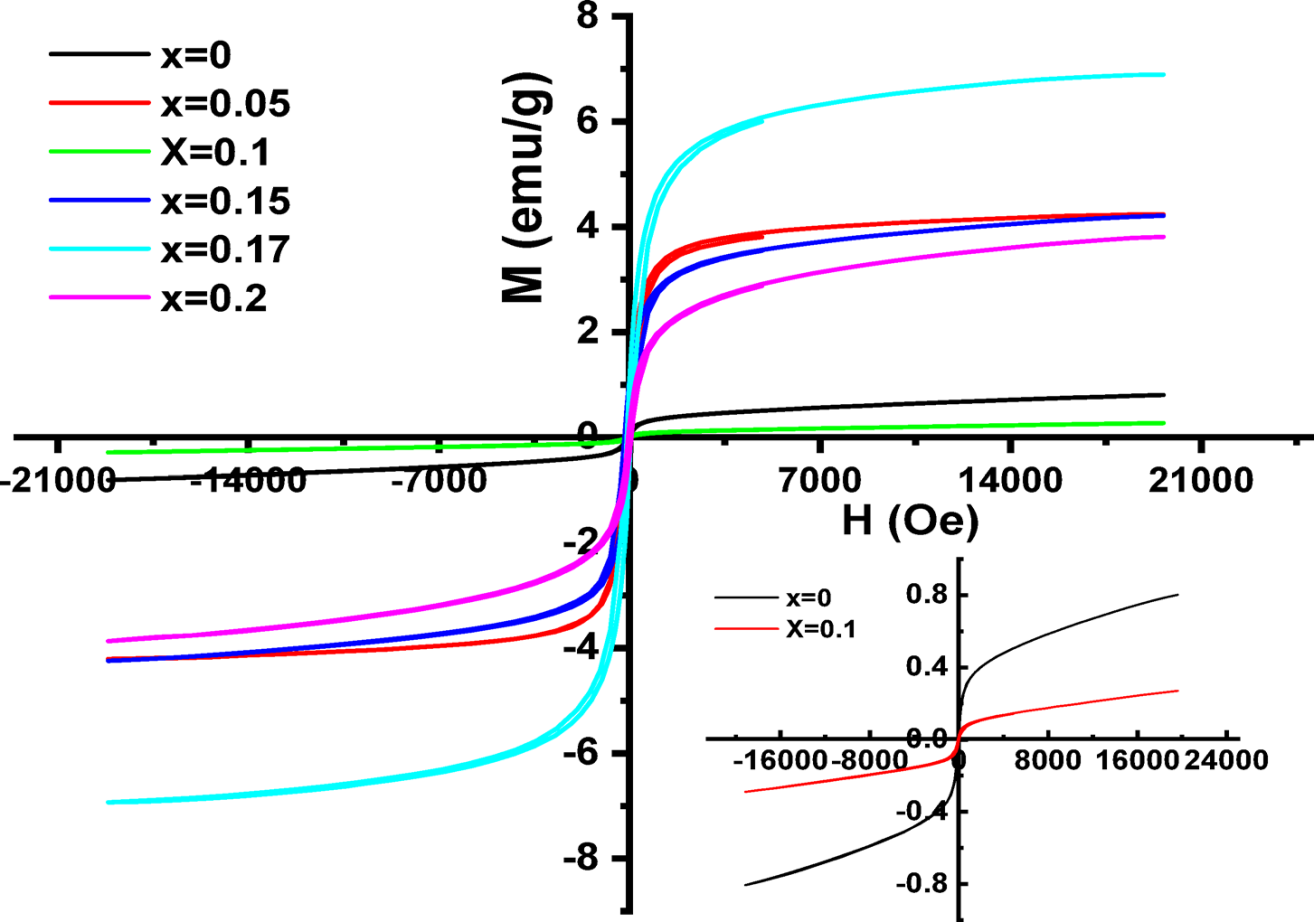
The M(H) hysteresis loop for La_1 − x_Mg_x_FeO_3_ samples.

The magnetization curves obtained from (VSM) measurements at room temperature for all La_1 − x_MgₓFeO₃ samples are shown in Fig. 8. Key magnetic parameters, including saturation magnetization (Ms), remnant magnetization (M_r_), and coercivity (Hc), were extracted and are summarized in Table 5. These parameters exhibit enhancement with increasing Mg doping, but the trends are non-monotonic, indicating complex magnetic behavior.

Among all samples, the composition with x = 0.17 displays the highest saturation magnetization, while the undoped (x = 0.00) and x = 0.10 samples exhibit the lowest values an unexpected outcome (see inset in Fig. 8). The hysteresis loops show clear inflections for x = 0.05, 0.15, 0.17, and 0.20 at%, suggesting the presence of room-temperature ferromagnetic (RT-FM) ordering. In contrast, samples with x = 0.0 and 0.10 exhibit weak ferromagnetism. This is particularly noteworthy, as bulk LaFeO₃ is typically known to be antiferromagnetic due to superexchange interactions between neighboring Fe³⁺ ions through Fe³⁺-O²⁻-Fe³⁺ linkages. The emergence of ferromagnetism in our nanoparticles can be attributed to several factors: Surface spin canting and uncompensated spins. At the nanoscale, the large surface-to-volume ratio leads to a high density of surface defects and uncompensated spins, which contribute to net magnetization. Similar surface-driven magnetic effects have been reported in LaFeO₃^80^, BiFeO₃^81,82^ and YFeO₃^83^.

Double exchange (DE) interaction: The coexistence of Fe³⁺ and Fe²⁺ ions may promote DE interactions, enhancing ferromagnetic coupling. This mechanism has been widely proposed in doped transition metal oxides exhibiting RT-FM. Exclusion of transition metal (TM) clusters. Although TM clusters such as Fe, FeO, or Fe₂O₃ are known to influence magnetic behavior, their contribution in this study is unlikely^84–86^ The observed magnetic moment (M = 0.28 emu/g) is significantly lower than values reported for LaFeO₃ with known TM clustering, including 0.38 emu/g for sol-gel LaFeO₃ nanoparticles (~ 21.9 nm)^87^ 0.44 emu/g for ball-milled samples (~ 50 nm)^88^, and 0.90 emu/g for electrospun nanofibers (~ 20 nm)^89^ If TM clusters were present, a much higher magnetization would be expected, proportional to the TM content. The variation in M_s_ values across studies may be attributed to differences in synthesis method, particle size, surface disorder, and oxygen vacancy concentration. The highest values of M_s_, M_r_, and H_c_ observed for x = 0.17 may result from enhanced surface spin effects and stronger Fe³⁺/Fe⁴⁺ interactions in the near-surface region. Overall, the magnetic behavior confirms the presence of ferromagnetic and weak ferromagnetic ordering in all samples, with no evidence of superparamagnetism. To further confirm the DE mechanism, complementary techniques such as (ESR) should be employed.

The variation in coercivity (H_c_) among the La_1 − x_Mg_x_FeO₃ samples can be attributed to differences in magnetocrystalline anisotropy. The anisotropy constant (K) was calculated using the following relation^90^.

5 $$K = {{\left[ {H_{c} \times {\text{ }}M_{s} } \right]} \mathord{\left/ {\vphantom {{\left[ {H_{c} \times {\text{ }}M_{s} } \right]} {0.96}}} \right. \kern-\nulldelimiterspace} {0.96}}$$

Additionally, the magnetic moment (µ_B_, in Bohr magnetons) was estimated using the formula:

6 $$\mu _{B} = {\text{ }}{{\left[ {M_{w} \times {\text{ }}M_{s} } \right]} \mathord{\left/ {\vphantom {{\left[ {M_{w} \times {\text{ }}M_{s} } \right]} {5585}}} \right. \kern-\nulldelimiterspace} {5585}}$$

where M_w_ is the molecular weight of the respective sample, and M_s_ is the saturation magnetization. The variation in µ_B_ can be linked to structural disorder such as lattice defects, internal strain, or deviations in long-range crystallinity. These structural anomalies contribute to differences in the magnetic behavior of the samples compared to what has been previously reported in the literature^91^. Changes in the crystal structure affect the bond length, bond angle, and bandwidth between transition metal cations and oxygen anions, as shown in Tables 3 and 4. Such modifications directly influence the strength of magnetic interactions.

In particular, the double exchange (DE) interaction is enhanced as the Fe-O-Fe bond angle decreases. This angular distortion arises from the substitution of La³⁺ (ionic radius ≈ 1.032 Å) with the smaller Mg²⁺ ion (0.72 Å), which introduces lattice compression. The decrease in bond angle becomes more pronounced at higher Mg doping levels, suggesting a corresponding increase in DE-mediated ferromagnetic coupling. Furthermore, the observed magnetic phase transitions may resemble those associated with the Griffiths phase in manganites^92^, wherein ferromagnetic clusters form within a predominantly paramagnetic matrix. Such magnetic inhomogeneity can be detected using (ESR) spectroscopy, which reveals the presence of multiple magnetic environments as distinct peaks or split signals in the ESR spectrum.

**Table 5:** Magnetic parameters obtained from the M (H) curve for the La_1 − x_Mg_x_FeO_3_ samples.

Sample (at%)	X = 0.0	X = 0.05	X = 0.1	X = 0.15	X = 0.17	X = 0.2
M_s_ (emu/g)	0.804	4.2212	0.28	4.2264	6.9125	3.8381
M_r_ (emu/g)	0.00298	0.15967	0.00187	0.65866	0.80894	0.25605
H_c_ (G)	27.721	15.631	62.241	103.54	71.767	52.119
S	3.71E-02	3.78E-02	0.066623	0.15584	0.11703	6.67E-02
K	23.2	68.7	18.1	455.8	516.8	208.4
µ_B_	0.0349	0.1791	0.0116	0.1707	0.2763	0.1511
C.S (nm)	38.01426	35.0987	25.67666	37.61634	42.4479	33.9838

### Electron spin resonance (ESR)

**Fig. 9:**
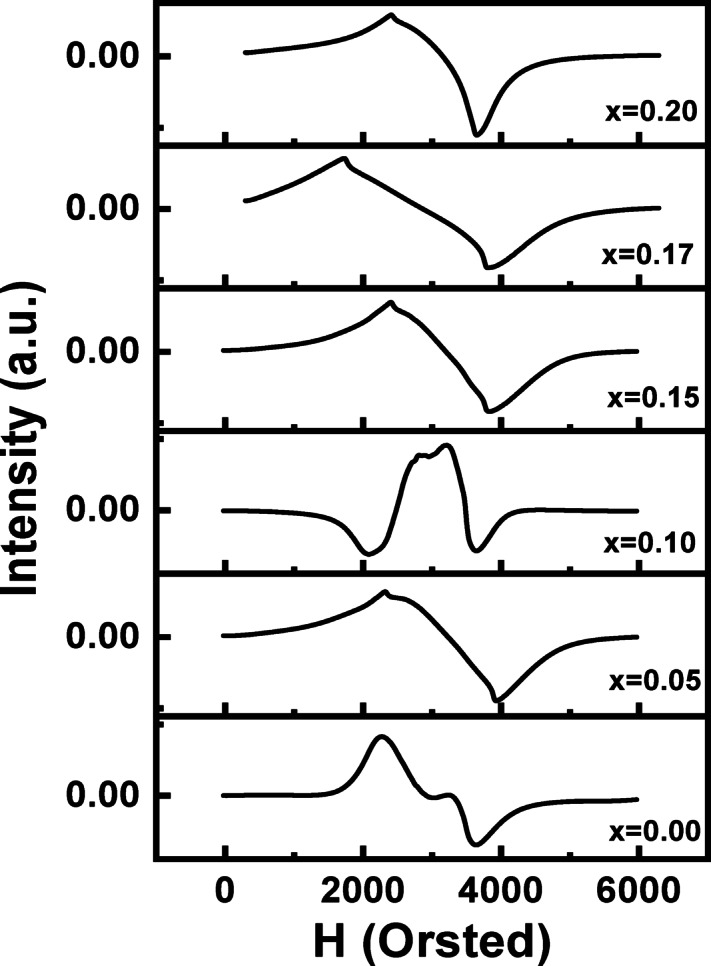
(ESR) spectroscopy for the La_1 − x_Mg_x_FeO_3_ samples.

(ESR) studies were conducted on all La_1 − x_Mg_x_FeO₃ samples to investigate their magnetic behavior at the microscopic level. Key ESR spectral parametersincluding line width (ΔH), effective g-factor, resonance field position (H_r_), and double-integrated intensity (DIN)were extracted and analyzed in detail from the spectra shown in Fig. 9.

The ESR resonance intensity (I_ESR_) is a critical parameter that provides insight into the nature and contribution of magnetic ions, such as Fe³⁺ and Fe⁴⁺, to the resonant species^93^ Fig. 9 displays the first derivative ESR spectra (dP/dH), which reveal characteristic ferromagnetic resonance (FMR) signals associated primarily with Fe³⁺ and Fe⁴⁺ spin dynamics. Each spectrum is defined by its resonance field, line width, and signal intensity.

Notably, all ESR spectra exhibit broad, asymmetric lines with two observable resonance peaks. This asymmetry is attributed to the random orientation of magnetic nanoparticles and their anisotropic crystal fields. The unbalanced line shapes and broadening of the signals are indicative of magnetic inhomogeneity, which is often caused by structural distortions such as variations in Fe-O-Fe bond angles and bond lengths, as well as changes in electronic bandwidth. The presence of multiple magnetic environments within the samples is also supported by the anisotropic axes of the nanoparticles. Such anisotropy leads to differing local magnetic fields, resulting in the observed asymmetric and broadened ESR profiles^94^.

Table 5 presents the ESR-derived values for the internal magnetic field (H_in_), resonance field (H_r_), peak-to-peak line width (ΔH_pp_), and Lande’s g-factor for the La_1 − x_MgₓFeO₃ samples. The data reveal significant variation in H_r_ values across different doping levels, which differ notably from the expected resonance field of isolated free electrons (H_r_ ≈ 340 mT, g ≈ 2.0023). This deviation indicates strong internal magnetic interactions between nanoparticles.

According to Sparks^95^ and Anderson^96^ the internal magnetic field (H_in_) significantly influences the effective magnetic resonance field. Three main sources contribute to the internal field in such nanoparticle systems:

(i) demagnetization effects.
(ii) dipole-dipole interactions between adjacent magnetic nanoparticles.
(iii) internal magnetic fields arising from particle agglomeration.

The effective magnetic field can be expressed as the sum of internal and external contributions:

7 $$Heff = Hext + Hin = constant$$

where H_ext_ is the externally applied field, measured as the resonance field (H_r_) by the ESR spectrometer, and H_in_ is the internal magnetic field.

As shown in Table 5, the variation in H_in_ values leads to resonance field shifts in either direction. Specifically, an increase in H_r_ corresponds to a decrease in H_in_, and vice versa. This inverse relationship supports the hypothesis that internal interactions-particularly between Fe³⁺ and Fe⁴⁺ ions, play a crucial role in shaping the magnetic response of the materials. The sample with x = 0.17 at% exhibits the strongest internal field, correlating with the highest saturation magnetization (M_s_), further confirming the impact of Fe³⁺/Fe⁴⁺ interactions on magnetic ordering.

**Table 6:** The values of the internal field (H_in_), g-factor, peak-to-peak line width (H_pp_), and resonance field (H_r_) derived from the ESR spectra for the La_1 − x_Mg_x_FeO_3_ samples.

Sample	0	0.05	0.1	0.15	0.17	0.2
H_o_ (mT)	309.33	318.13	348.04	314.02	272.66	307.85
∆H (mT)	135.18	162.06	44.27	140.71	206.32	124.11
H_in_ (mT)	30.67	21.87	−8.04	25.98	67.34	32.15
g-value	2.238	2.1761	1.9891	2.4322	2.539	2.2487

Moreover, Jung-Hoon Jeong et al.^97^ demonstrated that changes in H_r_ can also be linked to grain size, suggesting that microstructural features such as particle size and boundary effects influence ESR parameters.

The Lande g-factor and peak-to-peak line width (ΔH_pp_) offer additional insight into the nature of magnetic interactions.

The g-factor can be calculated using the following equation:

8 $$g = {{h\upsilon } \mathord{\left/ {\vphantom {{h\upsilon } {\mu _{B} H_{r} }}} \right. \kern-\nulldelimiterspace} {\mu _{B} H_{r} }}$$

where h is Planck’s constant, ν is the microwave frequency, µ_B_ is the Bohr magneton, and H_r_ is the resonance field.

As indicated in Table 5, the g-values vary significantly with Mg content, deviating from the free electron value (g = 2.0023), which suggests that the Fe³⁺ and Fe⁴⁺ ions are magnetically coupled rather than isolated. Higher g-values and broader line widths generally reflect increased dipolar interactions and reduced superexchange coupling^89^. This behavior confirms that the strength of dipole-dipole interactions increases with Mg doping, while superexchange interactions become less dominant. The sample x = 0.1 exhibits a low saturation magnetization compared to the whole samples; this may be attributed to defect-induced magnetic frustration that is directly supported by both VSM and structural (XRD) data, and microscopically justified by the nature of the (ESR) signals. The VSM measurements explicitly indicate a non-monotonic trend where the x = 0.10 sample exhibits one of the lowest Ms values, comparable only to the undoped x = 0.0 sample. This macroscopic collapse of magnetization correlates with a critical structural inflection point. The unit cell volume, which increased from the pristine sample (60.5337 Å^3^) to a maximum at x = 0.05 (60.6780 Å^3^ due to lattice expansion from Fe^2+^ creation and oxygen vacancies, then sharply drops at x = 0.10 (60.538Å^3^), nearly returning to the undoped value. This structural reversal suggests that x = 0.1 represents a threshold where defect chemistry shifts, causing a maximum concentration of oxygen vacancies and structural disorder4. These oxygen vacancy defects act as disruption sites by interrupting the Fe-O-Fe paths, severely suppressing the fundamental super exchange, and preventing the formation of the Fe^3+^- Fe^4+^ double-exchange (DE) paths necessary for strong ferromagnetism. Microscopically, this state of weak coupling is reflected in the ESR results: the low Ms x = 0.1 is correlated with a relatively narrow ESR linewidth and a weak dipolar interaction, which is characteristic of weakly coupled or frustrated spins, confirming that the substitution at this specific concentration fails to establish the collective, long-range magnetic order observed in the magnetically enhanced x = 0.17 sample. As doping proceeds past x = 0.10, the Fe^4+^ concentration increases, and the beneficial double-exchange mechanism overwhelms the disorder, leading to the sharp recovery and maximization of Ms at x = 0.17.

Finally, ESR parameters such as g-factor and ΔH_pp_ can be influenced by structural factors, including morphology, porosity, and degree of crystallinity, all of which are sensitive to doping and synthesis conditions. These ESR results are consistent with the VSM findings and further confirm the presence of weak and intermediate-range ferromagnetic interactions across all doping levels.

## Conclusion

In this study, Mg-doped LaFeO₃ (La_1 − x_MgₓFeO₃) perovskite nanoparticles were successfully synthesized using a facile green synthesis method. The structural analysis confirmed the formation of both cubic and orthorhombic phases depending on the doping concentration and synthesis conditions. XRD and Rietveld refinement analysis results revealed that Mg²⁺ ions were effectively incorporated into the LaFeO₃ lattice, inducing slight distortions in the FeO₆ octahedra and modifying the bond lengths and bond angles, as observed in both cubic and orthorhombic structures.

Dielectric measurements showed that Mg doping enhanced the dielectric constant while reducing dielectric loss, indicating improved polarization behavior and reduced energy dissipation, which are favorable for capacitor and energy storage applications. Magnetic analysis revealed that Mg substitution disrupts the antiferromagnetic ordering of pure LaFeO₃, leading to weak ferromagnetic behavior due to the introduction of lattice strain and modification of Fe-O-Fe superexchange pathways.

Overall, this work highlights the potential of Mg-doped LaFeO₃ perovskites as multifunctional materials with tunable structural, dielectric, and magnetic properties. The use of a green, low-cost synthesis method further supports the development of environmentally friendly materials for electronic, magnetic, and energy-related applications.

## Data Availability

The datasets used and/or analysed during the current study are available from the corresponding author on reasonable request.
